# Seismic fragility analysis of fully prefabricated frame structures with steel plate hoop bolt connections based on various engineering demand parameters

**DOI:** 10.1371/journal.pone.0350096

**Published:** 2026-05-27

**Authors:** Zhiyuan Gao, Jiaolei Zhang, Lei Cao

**Affiliations:** 1 School of Civil and Transportation Engineering, Yellow River Conservancy Technical University, Kaifeng, China; 2 Guangdong Provincial Key Laboratory of Intelligent Disaster Prevention and Emergency Technologies for Urban Lifeline Engineering, Dongguan University of Technology, Dongguan, China; 3 College of Urban Development and Modern Transportation, Xi’an University of Architecture and Technology, Xi’an, China; China Construction Fourth Engineering Division Corp. Ltd, CHINA

## Abstract

Steel plates and bolted connections have become common construction details in prefabricated structural systems. However, prefabricated frame structures with steel plate hoop–bolted connections exhibit connection gaps, interface slip, and discontinuous force-transfer paths, making them prone to hysteretic degradation and cumulative damage under seismic loading. Conventional seismic fragility assessments typically rely on the maximum inter-story drift ratio, which focuses only on deformation demand and fails to capture key deterioration mechanisms such as low-cycle damage accumulation, stiffness degradation, and reduced energy dissipation capacity. Consequently, the seismic performance of prefabricated structures may be inadequately represented. To address this limitation, this study adopts a two-parameter damage model as the engineering demand parameter for incremental dynamic analysis (IDA) and fragility assessment, and compares it with the traditional drift-based index. An energy-dissipation-based story damage weighting method is further introduced to better characterize damage distribution and performance degradation along the structural height. Finite element models of prefabricated columns and beam–column joints with steel plate hoop–bolted connections were developed in SAP2000 using multilinear plastic link elements and validated against quasi-static test results. A comparative fragility analysis was then performed for six-story full prefabricated and cast-in-place frame structures. The results show that the inter-story drift ratio underestimates structural capacity in the elastic stage but overestimates collapse resistance compared with the two-parameter damage model.

## 1. Introduction

Driven by the global advancement of construction industrialization and the rising implementation of green building initiatives in urban development, prefabricated construction has gained continuous policy support and technological impetus. Prefabricated structures, combining factory-made components with rapid on-site assembly, can significantly shorten construction time, reduce costs, and minimize construction waste and environmental impact, making them an important approach for modern industrialized and sustainable construction [1]. In areas with frequent earthquakes, prefabricated structures that can maintain reliable seismic performance while improving construction efficiency are not only easier to implement in practice but also play a key role in enhancing building safety and structural resilience.

However, assessing the seismic safety of prefabricated frame structures presents particular challenges. Compared with traditional cast-in-place reinforced concrete frames, prefabricated structures have more complex joint connections, where interface slip and insufficient energy dissipation through steel plates may occur [2]. These factors can lead to local stiffness degradation and accumulation of damage under seismic loading, resulting in overall structural degradation that is more complex than in conventional cast-in-place frames. Currently, most seismic fragility analyses of prefabricated frames still rely primarily on a single deformation-based index, such as the maximum inter-story drift ratio [3–5]. Although such indices are convenient for engineering applications, they cannot fully capture the key degradation processes in prefabricated structures, including reduced overall energy dissipation and cumulative damage caused by declining joint performance. Therefore, relying solely on a single deformation-based parameter for seismic assessment may underestimate the potential earthquake-induced damage of prefabricated structures.

Although research on the seismic fragility of prefabricated and cast-in-place frame structures has made progress, several limitations remain. For instance, Srikanth and Borghate [6] performed incremental dynamic analyses and developed fragility curves for precast concrete frames, but the study mainly used the maximum inter-story drift ratio as the demand parameter, ignoring the effect of cyclic energy dissipation on damage accumulation. Yahyaabadi et al. [7] considered joint nonlinearity in the fragility assessment of prefabricated frames, yet peak deformation was still used to define damage, and a two-parameter damage measure was not introduced, leaving energy-based damage mechanisms unquantified. Li et al. [8] studied multi-story prefabricated and cast-in-place frames and revealed collapse patterns, but the work focused on component-level failures and did not analyze how cumulative damage at joints affects overall structural performance. To address these issues, Feng et al. [9] proposed a probabilistic performance assessment based on a two-parameter damage model, confirming the limitations of single deformation indices and showing that the two-parameter model can provide a more complete evaluation of seismic performance. However, the method is mostly applied to conventional cast-in-place structures, and its applicability and practical validation for prefabricated frames with complex joints remain limited. To this end, Cao et al. [10] investigated damage evolution in prefabricated beam–column joints under cyclic loading and quantified damage using a modified Park–Ang two-parameter model, but the research focused on local joint performance, such as stiffness degradation and ultimate strength, without extending to overall structural fragility. In contrast, Zhang et al. [11] applied the two-parameter damage model to prefabricated column connections and showed that using only the maximum inter-story drift ratio may underestimate the fragility of connections caused by reduced energy dissipation, indicating that the true seismic risk of prefabricated structures could be overlooked. In summary, the main limitations of current studies are: (1) demand indices are still primarily based on peak displacement and cannot systematically capture cumulative damage driven by energy dissipation; (2) the application of two-parameter damage models in prefabricated structures remains limited. The main contribution of this paper is that, based on a two‑parameter damage model, it quantifies the additional damage caused by energy dissipation on top of traditional displacement‑based indicators. Moreover, to account for the varying importance of different stories, the damage at each story is weighted, thereby logically providing a more reasonable engineering demand parameter for structural fragility assessment.

To address these challenges, this study proposes a seismic fragility assessment method for prefabricated frame structures based on a two-parameter damage model. The method adopts a two-parameter damage model that accounts for both deformation demand and energy dissipation as the structural damage demand parameters, and introduces a floor-level damage weighting approach based on energy dissipation contribution to perform IDA and complete the seismic fragility assessment. The results from the two-parameter damage model are also compared with those obtained using the traditional maximum inter-story drift ratio to reveal differences in structural performance assessment under different indicators, thereby providing a more realistic representation of damage accumulation and overall performance degradation in prefabricated structures under strong seismic excitations. To verify the practicality of the presented approach, a frame structure composed of prefabricated beam–column and column–column connections using steel plate hoops and bolts was established and analyzed for seismic fragility. Some forms of this connection system have already been adopted in practical engineering applications. However, the corresponding structural systems have not yet been verified under strong earthquake excitations. Thus, a systematic seismic fragility analysis is required to evaluate the seismic fragility and offer a theoretical basis for the seismic design and further application of such prefabricated structures. To improve the efficiency of seismic response analysis, simplified numerical models of the prefabricated column–column and beam–column joints were developed in SAP2000 using multi-linear plastic link elements, and their accuracy was validated through quasi-static tests on the joints. Based on this, a six-story full prefabricated reinforced concrete frame and its corresponding cast-in-place frame were selected for comparative analysis.

## 2. Methodology

### 2.1. Analysis process

This study is devoted to developing a seismic fragility assessment framework for prefabricated frame structures, which includes the following steps. (1) Selection of structural damage indicators and weighting method, in which a two-parameter damage model considering both deformation demand and energy dissipation capacity is adopted, and a floor-level damage weighting approach based on energy dissipation contribution is applied to quantify damage distribution across floors. (2) Model development and joint simplification, in which a frame composed of prefabricated beams–columns and columns connected by steel plate hoops and bolts is modeled, and simplified numerical models of column–column and beam–column joints are developed in SAP2000 using multi-linear plastic link elements to improve the efficiency of seismic response analysis. (3) Model validation, where the numerical models are validated against quasi-static tests to ensure accurate representation of joint nonlinear behavior and energy dissipation characteristics. (4) Case analysis and performance evaluation, in which IDA is performed to evaluate the seismic response and fragility of the structure, and the results from the two-parameter damage model are compared with those based on the conventional maximum inter-story drift ratio to reveal differences in structural performance assessment. This framework enables a full and physically consistent description of cumulative damage evolution and global performance deterioration in prefabricated frame structures subjected to intense seismic actions.

### 2.2. Two-parameter damage model

Under seismic loading, the damage level of a structure or component is influenced not only by the instantaneous deformation but also by its energy dissipation capacity. Therefore, the two-parameter damage model can be used to quantify the contributions of both deformation and energy dissipation to damage. It should be noted that the two-parameter damage model referred to in this study differs from conventional single-parameter models (e.g., inter-story drift ratio) in that it incorporates both displacement and energy-based components. The damage index *D* derived from the two-parameter model is generally bounded within the interval [0,1], where D = 0 indicates an intact structure or component, D = 1 represents complete failure, and 0 < *D* < 1 corresponds to intermediate states between intact and fully damaged [12]. For reinforced concrete components, the two-parameter damage model quantifies damage by integrating peak deformation response and cumulative hysteretic energy dissipation, thereby capturing the combined effects of instantaneous deformation and energy dissipation on component failure. Park and Ang [13] first proposed a linear combination form of the two-parameter damage model, which is expressed as follows:


$$\begin{array}{c} D=\frac{\delta_{m}}{\delta_{u}}+\beta \frac{\int dE}{P_{y}\delta_{u}} \end{array}$$ (1)

where *D* denotes the damage index; *δ*_m_ represents the peak deformation response of the member under seismic excitation; *δ*_u_ stands for the ultimate deformation capacity of the member under monotonic loading; *P*_*y*_ refers to the yield load of the member; ∫dE denotes the cumulative hysteretic energy; and *β* is the cyclic load influence factor, which varies between 0 and 0.85 with a mean value ranging from 0.10 to 0.15, and is calculated as follows:


$$\begin{array}{c} \beta =(-0.447+0.073\lambda +0.24n_{\text{0}}+0.314\rho_{s})\times 0.{\text{7}}^{\rho_{w}} \end{array}$$ (2)

where *λ* denotes the shear span ratio, which is taken as 1.7 when it is less than 1.7; *n*_0_ is the axial compression ratio, taken as 0.2 when *n*_0_ ＜ 0.2; *ρ*_s_ represents the longitudinal reinforcement ratio and *ρ*_w_ denotes the volumetric stirrup ratio.

Subsequently, Kunnath [14] modified this model to more accurately describe damage accumulation under cyclic loading within the nonlinear stage (see Eq. (3)).


$$\begin{array}{c} D=\frac{\delta_{m}-\delta_{y}}{\delta_{u}-\delta_{y}}+\beta \frac{\int dE}{P_{y}\delta_{u}} \end{array}$$ (3)

where *δ*_*y*_ represents the deformation of the component at yielding.

The two-parameter damage model presented in Eq. (3) has the advantage of considering both the peak deformation response and cumulative hysteretic energy, and it has been widely applied in component-level in previous studies. However, for an entire frame structure, calculating cumulative hysteretic energy typically requires summing the contributions of numerous beams and columns, which is computationally intensive and complex, making direct application in engineering practice challenging and limiting the model’s broader use. Therefore, it is necessary to simplify the calculation of cumulative hysteretic energy for frame structures. To address this issue, this study adopts the approach proposed in [15], establishing a relationship between floor-level hysteretic energy and structural maximum displacement from an energy perspective. The correspondence between the elastic–plastic deformation energy at each floor and the structural maximum displacement, as proposed in the literature, is expressed as follows:


$$\begin{array}{c} E=\frac{\text{1}}{\text{2}}F_{y}x_{y}+(1+4\rho )F_{y}(x_{m}-x_{y}) \end{array}$$ (4)

where *E* represents the elastic–plastic deformation energy of a given floor; *F*_y_ and *x*_y_ denote the interstory yield shear force and yield displacement, respectively; *ρ* is the hysteretic model correction factor, which is recommended as 0.33 in the case of reinforced concrete members [15]; and *x*_m_ refers to the peak elastoplastic displacement of the structure subjected to seismic loading. In this paper, the peak displacement of the structure under seismic action required in Eq. (4) is obtained using the SAP2000 software, while the yield displacement and yield force can be determined by the Pushover analysis method. Since the example in this paper uses connection elements in SAP2000 to replace plastic hinge models, this means that the nonlinear deformation and energy dissipation at the component level are mainly concentrated in the connection elements.

Ultimately, the damage index for each floor can be calculated using Eqs. (3) – (4). However, these calculations only reflect the damage level of individual floors. To quantify the damage of the entire frame structure, the damage indices of all floors need to be appropriately weighted and combined, as the consequences of failure at the top floor differ significantly from those at the bottom floor. This weighted approach allows for a more comprehensive representation of the overall structural performance degradation under seismic loading.

### 2.3. Energy consumption weighting method of different floors

The two-parameter damage model introduced in the previous section quantifies structural damage by considering both peak displacement response and cumulative hysteretic energy, capturing the effects of instantaneous deformation and energy dissipation under seismic loading. However, for mid-to high-rise prefabricated frame structures, evaluating damage solely at the component or section level is computationally demanding and does not adequately represent the overall structural performance. To overcome this limitation, a floor-level energy contribution weighting method is proposed, which extends the two-parameter damage indices from individual components to the global structural level. In this approach, the structure is divided into floor units, and the damage of each floor is weighted and aggregated to produce a quantified measure of the total structural damage. The weighting method builds on the strategies proposed by Park and Ang [13] (Eq. (5)) and is further improved here by introducing a new energy dissipation weighting formula (Eq. (6)). This formula accounts for differences in damage contribution among floors, assigning greater weight to floors that dissipate more energy, thereby providing a more realistic assessment of overall structural performance degradation under seismic loading.


$$\begin{array}{c} \eta_{i}=\frac{E_{h}}{\sum E_{h}} \end{array}$$ (5)

$$\begin{array}{c} \eta_{i}=\frac{\text{(}N-i+1\text{)}E_{h}}{\sum_{i=1}^{N}(N-i+1)E_{h}} \end{array}$$ (6)

where *η*_*i*_ represents the weighting factor and damage index of the *i*-th floor, respectively; and *N* is the total number of floors.

### 2.4 Seismic fragility assessment method based on the two-parameter damage model

Using the previously described two-parameter damage indices and floor-level energy contribution weighting method, this study establishes a seismic fragility evaluation method for prefabricated structures. The framework adopts the overall structural damage index *D* as the engineering demand parameter (*EDP*), capturing both the maximum deformation demand and the cumulative hysteretic energy effects under seismic loading, which provides greater physical reliability compared with conventional methods that characterize damage solely by inter-story drift. By employing a floor-level energy contribution weighting strategy, the approach maps local component damage to the global seismic behavior of the entire structure. Following the performance assessment concept proposed by PEER (Pacific Earthquake Engineering Research), this framework replaces the traditional displacement-based fragility demand parameter with an overall structural damage index derived from the two-parameter damage model, thereby establishing a probabilistic relationship between the seismic intensity measure (*IM*) and the structural damage index *D*. Under the assumption of a lognormal distribution, the probability that the structure reaches or exceeds a certain damage state under a given seismic intensity *IM* can be expressed as:


$$\begin{array}{c} P[D\geq D_{lim}|IM]=\Phi \left(\frac{ln\left(D\right)-ln\left(D_{lim}\right)}{\sqrt{\overset{\text{2}}{\underset{D|IM}{\beta }}+\overset{\text{2}}{\underset{C}{\beta }}}}\right) \end{array}$$ (7)

$$\begin{array}{c} \beta_{D|IM}=\sqrt{\frac{{\sum_{i=1}^{n}\left[ln\left(D_{i}\right)-ln\left(D\right)\right]}^{\text{2}}}{n-2}} \end{array}$$ (8)

where *D*_lim_ represents the threshold corresponding to a specific damage state (e.g., slight, moderate, severe, or collapse), which serves as a capacity parameter of the structure, *D* is the mean value of structural demand under a given ground motion intensity measure, *β*_D|IM_ and *β*_C_ denote the logarithmic standard deviations of structural demand and structural capacity, respectively, which are calculated according to Eq. (8), D*i* represents the structural demand obtained under the *i*-th ground motion record, and *IM* denotes the seismic intensity measure, such as the peak ground acceleration (PGA). To obtain the relevant parameters in Eq. (7), multiple relationships between the seismic intensity *IM* and the total structural damage *D* are obtained through IDA using amplified ground motion records. Based on previous studies, it is commonly assumed that these two quantities follow a log-linear relationship, which is fitted by the least squares method, as expressed in Eq. (9).


$$\begin{array}{c} lnD=a+b\cdot ln\left(IM\right) \end{array}$$ (9)

where *a* and *b* are fitting parameters.

## 3. Numerical modeling and Test validation

Based on the analysis framework presented in Section 1, once the performance evaluation index of the prefabricated frame structure is defined—namely, the damage quantified by the proposed two-parameter damage model—it is still necessary to simplify the modeling of structural joints to enable efficient analyses under multiple seismic excitations. To calibrate these finite element simplifications, quasi-static tests were carried out on prefabricated beam–column joints and column joints. Test data were adopted to verify and optimize the simplified models, so as to guarantee their accuracy and reliability in the subsequent seismic fragility analysis.

### 3.1. Test program

The design parameters and basic experimental information of the prefabricated beam–column specimens and column specimens are presented in sequence below.

#### 3.1.1. Design of the prefabricated beam–column specimens.

**Fig 1:**
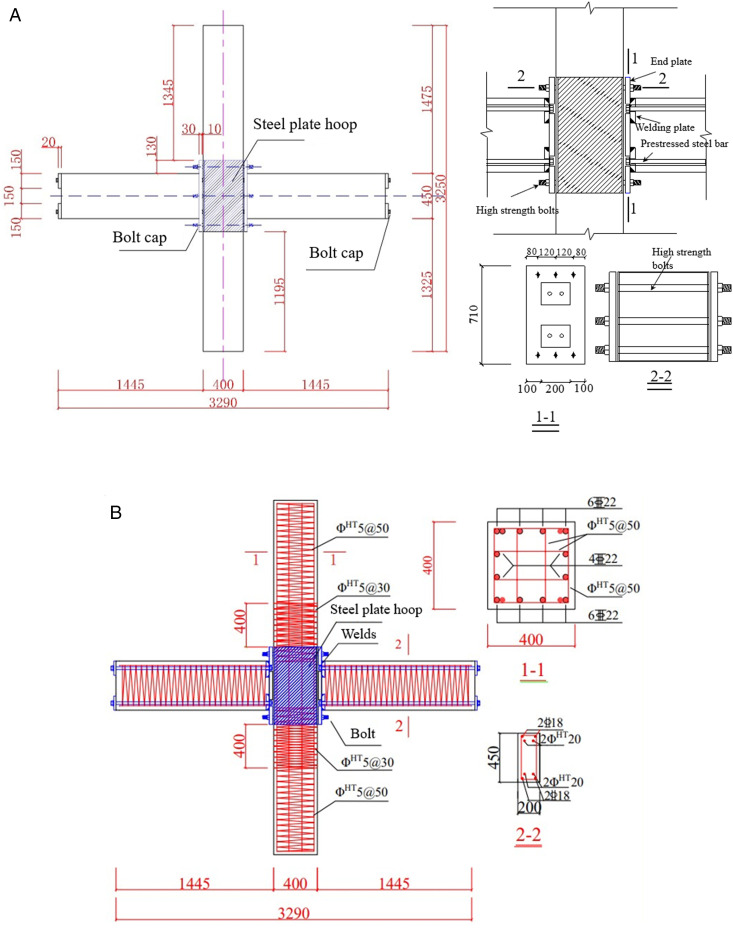
Fabricated beam-column specimen structure diagram. (a) Fabricated beam-column joint structure diagram. (b) Reinforcement diagram of the specimen.

A total of six prefabricated beam–column specimens with steel plate hoop and bolt connections were designed, labeled #PAN-01 to #PAN-06. The stirrup arrangements included single-layer hoops (#PAN-01 to #PAN-04) and double-layer hoops (#PAN-05 and #PAN-06). All columns were made of C40 concrete with a cross-section of 400 mm × 400 mm. The longitudinal reinforcement consisted of 22 mm diameter HRB600 bars, while the stirrups were 5 mm diameter HTH1100 high-strength continuous composite spiral hoops with a regular spacing of 50 mm and a reduced spacing of 30 mm in the confinement zone. To facilitate assembly and improve confinement in the joint region, 4 mm thick steel plate hoops were used instead of internal column stirrups. The beams were made of C35 concrete with a cross-section of 200 mm × 450 mm. Longitudinal reinforcement included 18 mm diameter HRB400 bars and four 20 mm diameter HTH1080 high-strength prestressing strands. The detailed reinforcement information of all specimens can be referred to Table 1. The end plates had dimensions of 30 mm × 400 mm × 710 mm and were made of Q390B steel. The prefabricated beams and columns were connected using six 28 mm diameter HTH1080 high-strength bolts. Prestressing of the beam strands and high-strength bolts was applied manually using a 2000 N•m torque wrench, with the beam prestress set to 1200 N•m. During the tests, the axial compression ratio of the specimens was maintained at 0.25. The geometric dimensions and reinforcement arrangements of all specimens are illustrated in Fig. 1.

**Table 1:** Detailed parameters of each specimen.

Specimen ID	Dimension /mm	Longitudinal rebar	Transverse rebar	Torque / *N*•m	Stirrup spacing	
					Regular spacing	Close spacing
#PAN-01	200 × 450 （*b* × *h*）	4Φ18HRB400 4Φ20HTH1080	HPB300	900	Φ10@110	Φ10@90
#PAN-02			HTH1100	1200	Φ5@60	Φ5@50
#PAN-03					Φ5@70	Φ5@60
#PAN-04					Φ5@90	Φ5@70
#PAN-05		4Φ18HRB400 4Φ20HTH1080 4Φ6HPB300			Φ5@90	Φ5@70
#PAN-06					Φ5@100	Φ5@80

#### 3.1.2. Design of prefabricated column specimens.

**Fig 2:**
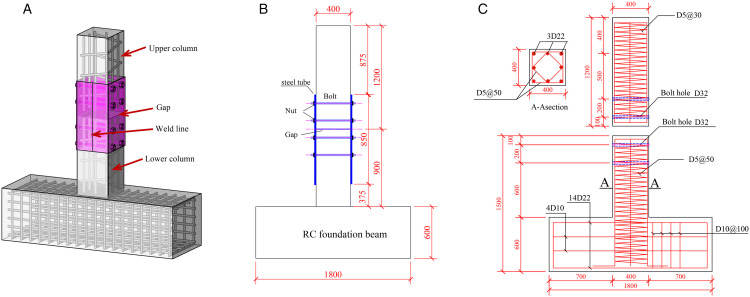
Fabricated column specimen structure diagram. (a) 3D drawing of prefabricated column (b) Prefabricated column diagram (c) PRCC-L01 and PRCC-L02.

As depicted in Fig 2a, the prefabricated reinforced concrete frame column adopts an integrated structural configuration, in which the full section is maintained at the column base, and the upper and lower precast segments are connected as a whole by steel plate hoops and bolts. The column is fabricated in two segments at the mid-height position, enabling convenient on-site assembly through bolted connections after aligning the upper and lower components. High-strength spiral hoops are also configured to confine the core concrete, aiming to guarantee favorable seismic performance. To investigate the seismic performance of the proposed connection, two specimens were designed and tested, with key test parameters listed in Table 2 and detailed dimensions shown in Fig 2b. The concrete used is of grade C40. Both specimens L01 and L02 are configured with eight HRB400 longitudinal bars of 22 mm diameter. High-strength spiral stirrups with a yield strength of 1100 MPa and a diameter of 5 mm are arranged at a spacing of 50 mm along the column height. A dense stirrup zone with 30 mm spacing is set within a 400 mm length near the column top. The cross-section of each column specimen is 400 mm × 400 mm.

**Table 2:** Parameters of specimens.

Specimen I.D.	*n*	λ	$\rho_{w}/\%$	*t*_w_/mm
PRCC-L01	0.2	4.5	0.79	8
PRCC-L02	0.6	4.5	0.79	5

Note: *n* denotes axial compression ratio; *λ* denotes shear span ratio; *ρ*_w_ denotes volume stirrup ratio; *t*_w_ denotes steel tube thickness.

### 3.2. Material properties

#### 3.2.1. Material properties of the prefabricated beam–column specimens.

The material properties of the precast beam–column specimens include the concrete materials used in both the beams and the columns. The concrete strength grades are C40 for the columns and C35 for the beams. According to the Chinese standard GB/T 50152−2012 [16], mechanical properties of the concrete were tested, and the results are summarized in Table 3. Tensile tests of the steel materials were carried out following the sampling requirements of GB/T228.1-2010 [17], in which three specimens were taken for each bar diameter. The samples were then machined into standard tensile coupons according to GB/T13239-2006 [18], and tested under the loading protocol specified in GB/T13239-2006 [18]. The corresponding mechanical properties are summarized in Table 4.

**Table 3:** Mechanical properties of concrete materials index.

Specimen	*f*_cu,k_/MPa	*f*_ck_/MPa	*E*_c_/MPa
Prefabricat column	41.9	28	3.3 × 10^4^
Prefabricated beam	28.4	19	2.92 × 10^4^

Note: *f*_cu,k_ stands for the standard cubic compressive strength of concrete, *f*_ck_ the standard axial compressive strength, and *E*_c_ the elastic modulus of concrete.

**Table 4:** Mechanical properties parameters of steel bar and steel plate.

Steel or rebar /mm	*f*_y_ / MPa	*f*_u_ / MPa	Elongation / %
5	1111	1337	2.83
6	541	682	8.24
10	377	560	10.36
18	448	631	12.47
20	1160	1230	12
22	698	878	21
28	1120	1180	14
−4	297	448	29

Note: *t* denotes the diameter of reinforcing steel bars or the thickness of steel plates. *f*_y_ represents the yield strength. *f*_u_ the ultimate strength, and *A* the elongation after fracture.

#### 3.2.2. Material properties of the prefabricated column specimens.

The designed compressive strength of concrete is grade C40. Material properties of concrete, reinforcing steel and steel tubes were determined following relevant Chinese codes, as summarized in Tables 5 and 6, and M4.8 grade bolts were adopted in this study. Specifically, concrete mechanical properties were tested in accordance with GB/T 50081−2019 [19], while material tests on steel products were performed according to GB/T 2975−2018 [20].

**Table 5:** Concrete properties.

Specimen I.D.	*f*_cu,k_/MPa	*f*_ck_/MPa	*E*_c_/MPa
PRCC-L01、PRCC-L02	43.76	29.27	3.34 × 10^4^

**Table 6:** Steel properties.

Steel or rebar /mm	*f*_y_/ MPa	*f*_u_ / MPa	*A*/%
22	490	662.5	24
14	462.5	642.5	19
5	1050	1170	—
—8	290	405	40

### 3.3. Loading scheme

For both the prefabricated beam–column and column specimens, load-controlled loading was applied before yielding, with one cycle per increment. After yielding, the loading was switched to displacement-controlled, with displacement increments equal to integer multiples of the yield displacement, and three cycles were applied at each increment. The loading continued until obvious failure occurred or the load dropped to 85% of the maximum load, at which point the test was terminated [21].

### 3.4. Development and validation of simplified models for prefabricated components

#### 3.4.1. Simplified model development.

**Fig 3:**
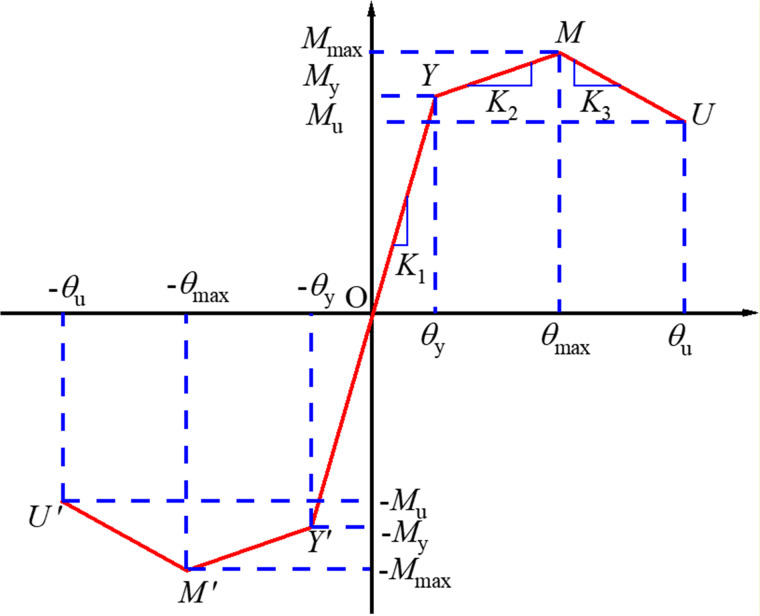
The stiffness values of prefabricated components at different failure stages.

Traditional solid-element modeling of prefabricated frame structures often encounters convergence difficulties and entails substantial computational costs in seismic response analysis [22,23]. To improve computational efficiency, this study employs SAP2000 to develop simplified models of prefabricated beam–column and column connections using multilinear plastic link elements, which release the rotational stiffness of the joint regions. The rotational stiffness at different loading stages is assigned along the *R*_1_ and *R*_2_ rotational directions of the nonlinear link elements to achieve stiffness equivalence, as illustrated in Fig 3. This approach is inspired by Hamutcuoglu [24] and Oksuz [25], who applied SAP2000 link elements to develop simplified bridge models for efficient seismic response analysis. The key points of the link elements, shown in Fig 3, include the yield point *Y* (*θ*_*y*_, *M*_*y*_), the peak point *M* (*θ*_*max*_, *M*_*max*_), and the ultimate point *U* (*θ*_*u*_, *M*_*u*_), with *K*_1_, *K*_2_, and *K*_3_ representing the stiffness in the elastic, hardening, and softening stages, respectively. The method for using this connection element specifically follows that proposed by Gao and Zhang [26]. In fact, the determination of the key parameters (K1, K2, K3, yield point, peak point, and ultimate point) has been completed in previous study [27–29], which has already provided the calculation models for these key points.

**Fig 4:**
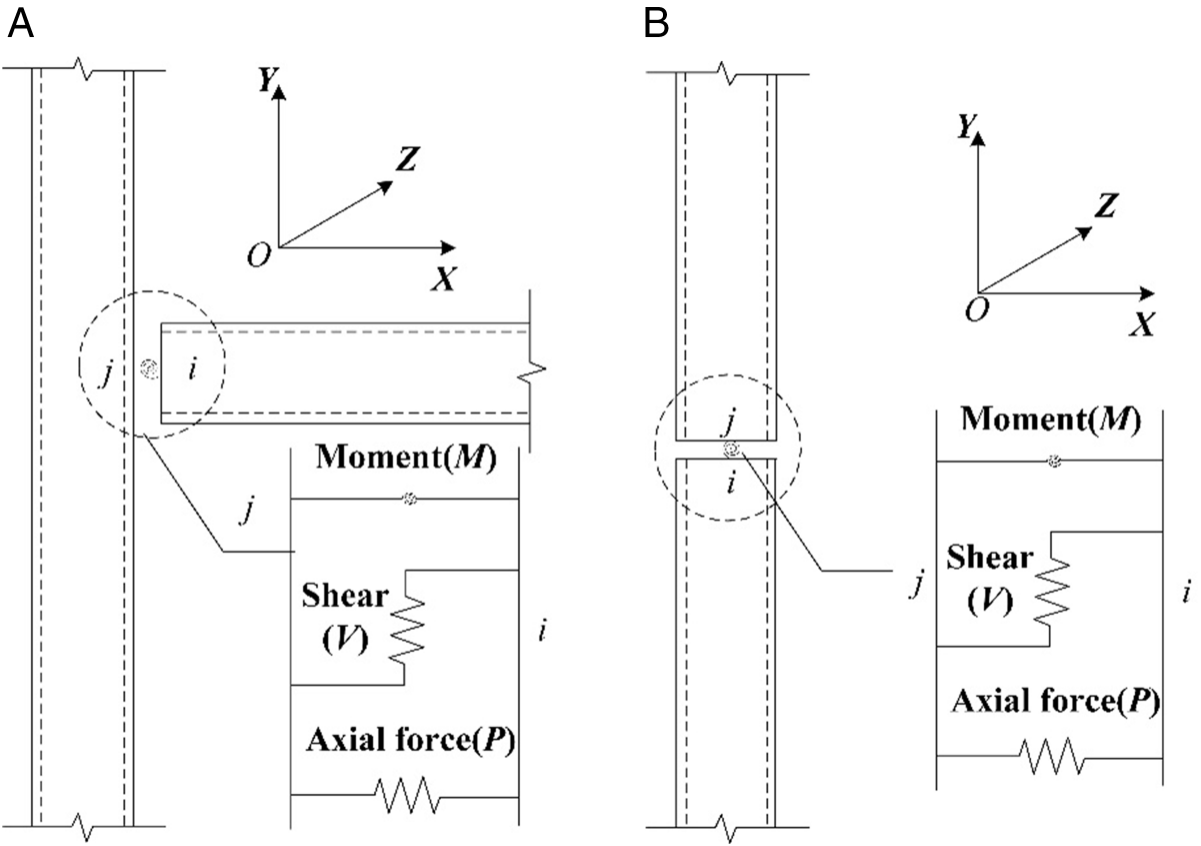
Simplified model diagram of prefabricated structure. (a) Simplified model diagram of beam-column joints (b) Simplified model diagram of column-column joints.

Each link element consists of six decoupled springs; as illustrated in the *XOY* plane in Fig 4, three springs represent axial deformation, in-plane shear deformation, and in-plane bending deformation. Taking Fig 4b as an example, point *i* is located at the lower column, and point *j* at the upper column, with the rotational degrees of freedom released at the *ij* nodes while translational degrees of freedom are constrained. Beams and columns are modeled using frame elements, and prestressing steel bars are represented by tendon elements. This modeling approach captures the effects of biaxial bending, torsion, axial deformation, and biaxial shear deformation. Each node of the element possesses six degrees of freedom: *U*_1_, *U*_2_, *U*_3_ for translations and *R*_1_, *R*_2_, *R*_3_ for rotations. The steel constitutive behavior is represented by a bilinear strain-hardening model, with the hardening stiffness set to 1/100 of the elastic stiffness [30]. Concrete behavior is simulated via the Mander model to characterize the stress–strain response of confined concrete [31]. The hysteretic behavior of the elements is defined using the Takeda multilinear hysteresis rule [32].

#### 3.4.2. Analysis of test results and verification of simplified model.

**Fig 5:**
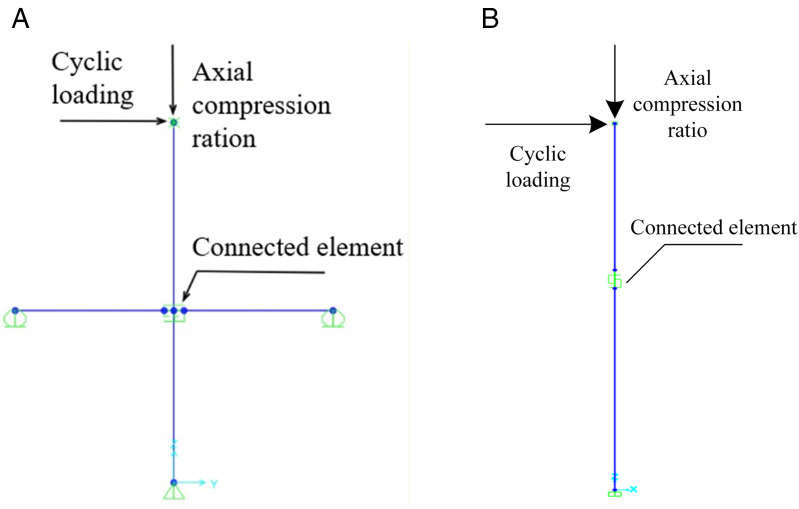
Simplified finite element model of prefabricated components. (a) Simplified model of prefabricated beam-column (b) Simplified model of prefabricated column.

**Fig 6:**
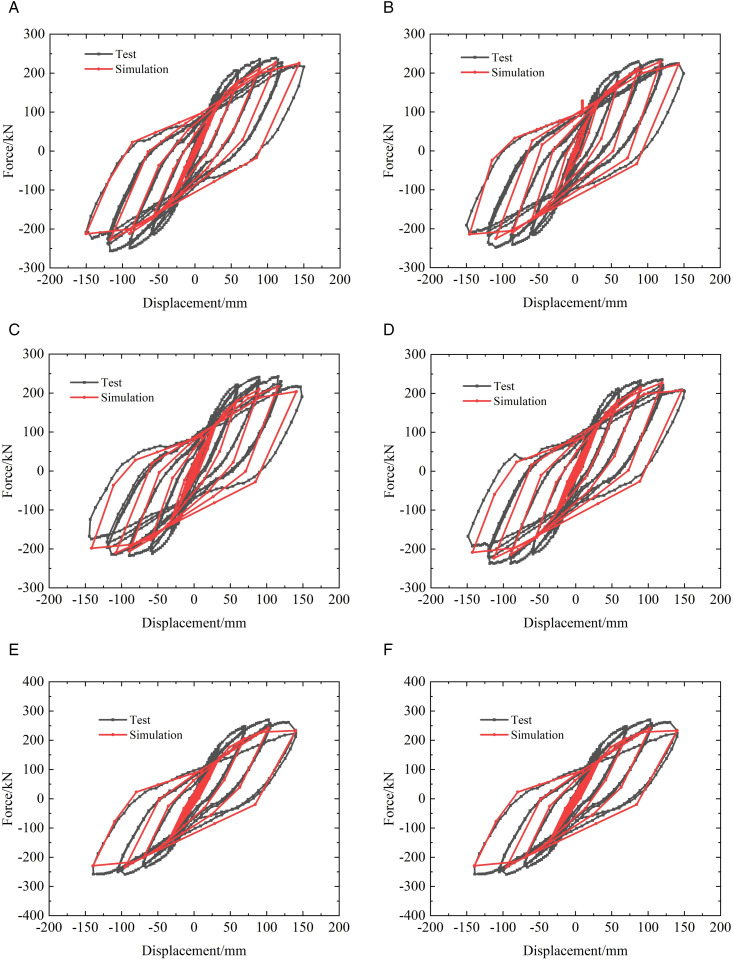
Verification of hysteretic curve of prefabricated beam-column specimens. (a) #PAN-01 (b) #PAN-02, (c) #PAN-03 (d) #PAN-04, (e) #PAN-05 (f) #PAN-06.

**Fig 7:**
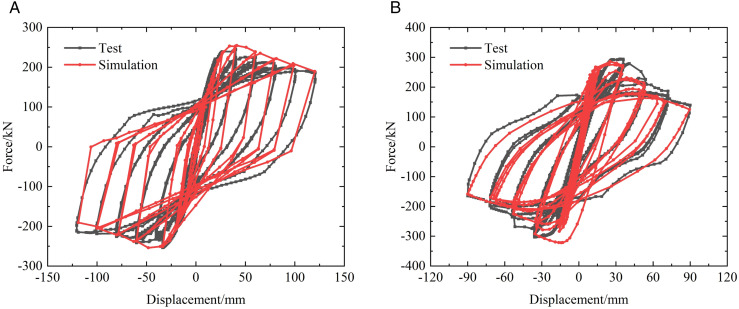
Verification of hysteretic curve of prefabricated column specimens. (a) PRCC-L01 (b) PRCC-L02.

To validate the proposed joint modeling strategy and assess the effectiveness of the nonlinear link elements, the prefabricated beam–column specimens and column–column specimens described in Section 2.1 were numerically simulated using the simplified finite element approach. The loading schemes adopted in the experiments and numerical analyses are illustrated in Fig 5, while the comparisons between the simulated and experimental hysteretic responses are presented in Figs 6 and 7.

From the experimental results, both the prefabricated beam–column and column specimens exhibit stable hysteretic behavior prior to severe damage, followed by gradual stiffness degradation and strength deterioration with increasing displacement amplitudes. The hysteresis loops show evident pinching and cumulative degradation, which are mainly associated with the slip and opening–closing behavior at the steel plate hoop–bolted connections, as well as the progressive yielding of steel components and concrete cracking. These characteristics indicate that the seismic response of the prefabricated joints is governed not only by peak deformation demand but also by cumulative cyclic effects. As shown in Figs 6 and 7, the simplified numerical models are able to reasonably reproduce the overall shape, strength envelope, and degradation trend of the experimental hysteretic curves for both types of specimens. In particular, the initial stiffness, peak load capacity, and post-yield stiffness degradation are well captured, demonstrating that the nonlinear link elements can effectively represent the dominant force-transfer mechanism and energy dissipation behavior of the prefabricated joints.

Nevertheless, certain discrepancies can be observed between the simulated and experimental responses, especially in the pinching degree and unloading stiffness. Previous studies on concentrated plasticity and link-based joint models have demonstrated that such approaches inevitably smooth local slip and contact effects into equivalent hysteretic parameters, leading to minor discrepancies in pinching severity and unloading stiffness when compared with experimental results [33,34]. However, considering the objective of achieving efficient and stable simulations under multiple seismic excitations, these discrepancies are acceptable and do not significantly affect the overall damage evolution or energy dissipation trends. Overall, the comparison results confirm that the proposed simplified joint modeling approach provides a satisfactory balance between computational efficiency and accuracy. It is therefore suitable for incorporation into the seismic fragility analysis of prefabricated frame structures, where repeated nonlinear time-history analyses are required.

## 4. Case study on seismic fragility assessment of a full precast frame structure

Once the simplified models of the prefabricated components are validated, they can be further used to develop equivalent models of prefabricated frame structures, enabling efficient structural analysis.

### 4.1. Structure information

**Fig 8:**
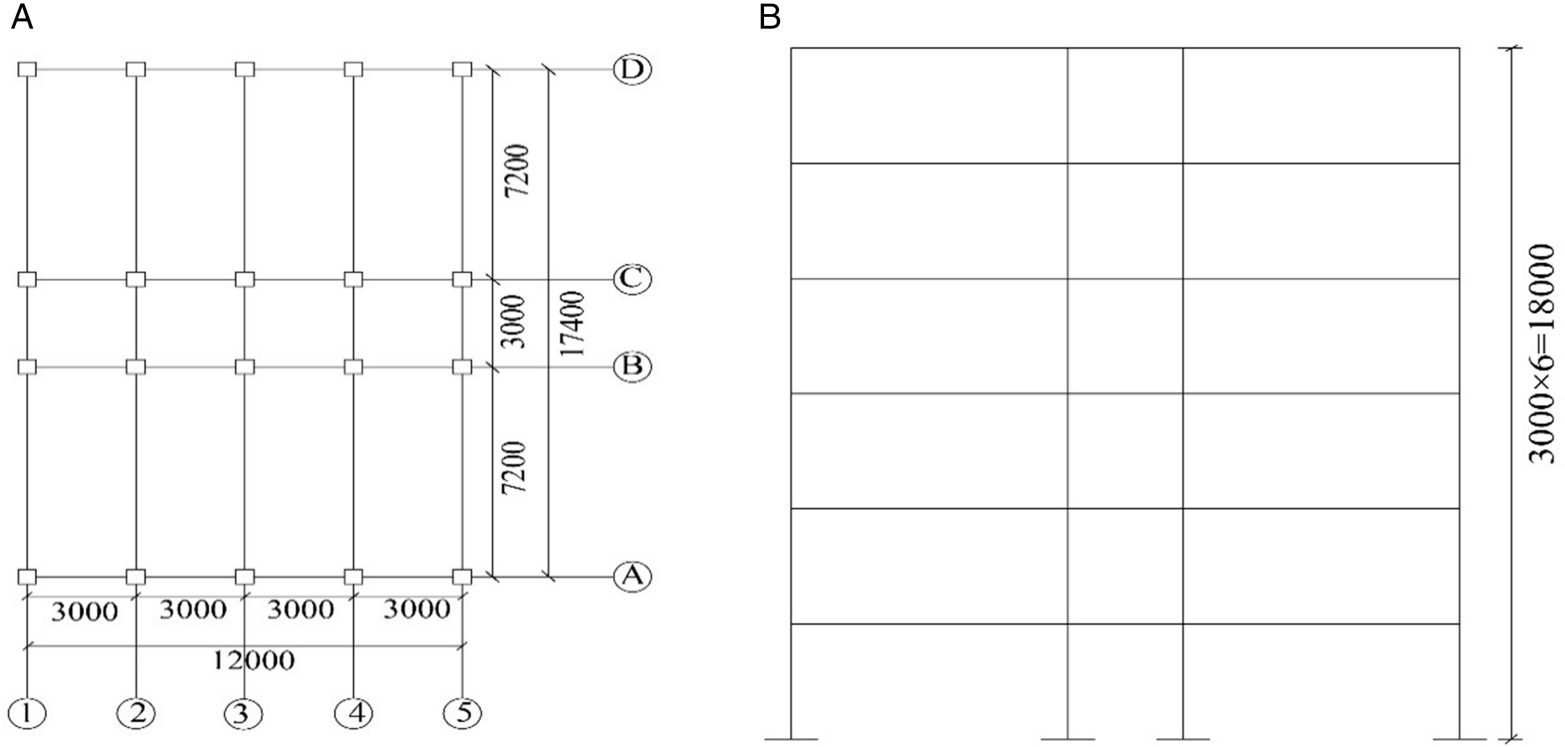
Prefabricated structure and cast-in-place structure design diagram. (a) Structural plane diagram (b) Structural elevation diagram.

**Fig 9:**
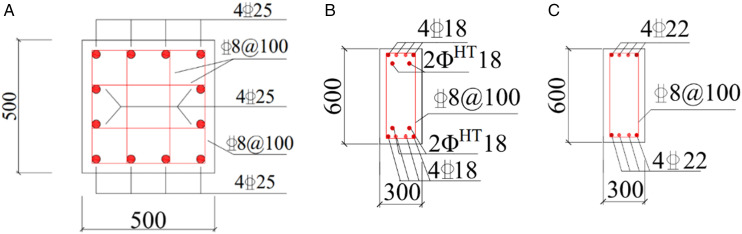
Reinforcement diagram of beam and column. (a) Column reinforcement (b) Prefabricated beam reinforcement (c) Cast-in-place beam reinforcement.

To demonstrate the seismic fragility assessment method proposed in this paper, the end-plate thickness and steel hoop thickness for the prefabricated beam–column joints were determined as 35 mm and 4 mm, respectively, based on experimental analyses of the prefabricated beam–column and column–column joints described in Section 2. The steel hoop thickness for the prefabricated column–column joints was set to 8 mm. Using these design parameters, a corresponding prefabricated RC frame model was developed. The structural design of the prefabricated frame followed the relevant specifications [35,36], and to enable a direct comparison of seismic performance, the member dimensions and reinforcement details were kept consistent with those of the cast-in-place counterpart. According to the Chinese design code [35], the structure belongs to the second seismic design group, with a site characteristic period of 0.4 s, Site Class II, a seismic fortification intensity of 8 degrees (0.2 g), and an importance factor of 1.0. All columns were designed with a cross‐section of 500 mm × 500 mm, and beams with a cross‐section of 300 mm × 600 mm. Beam and column members were modeled using frame elements. The concrete material followed the Mander confined concrete model, while the steel reinforcement was modeled using a bilinear hardening constitutive law. The applied loads were specified as follows: a roof dead load of 5 kN/m², roof live load of 0.5 kN/m², floor dead load of 4 kN/m², floor live load of 2 kN/m², beam line load of 9.6 kN/m, basic wind pressure of 0.35 kN/m², and basic snow pressure of 0.25 kN/m². The concrete strength grade was C40, ordinary longitudinal and transverse reinforcement adopted HRB400, and prestressing tendons used the high-strength heat-treated steel HTH1080 adopted in the Section 2 tests. The plan and elevation layouts of both the prefabricated and cast-in-place frame structures are shown in Fig 8, while the detailed beam–column cross-sections and reinforcement configurations are presented in Fig 9.

### 4.2. The selected ground motions

**Fig 10:**
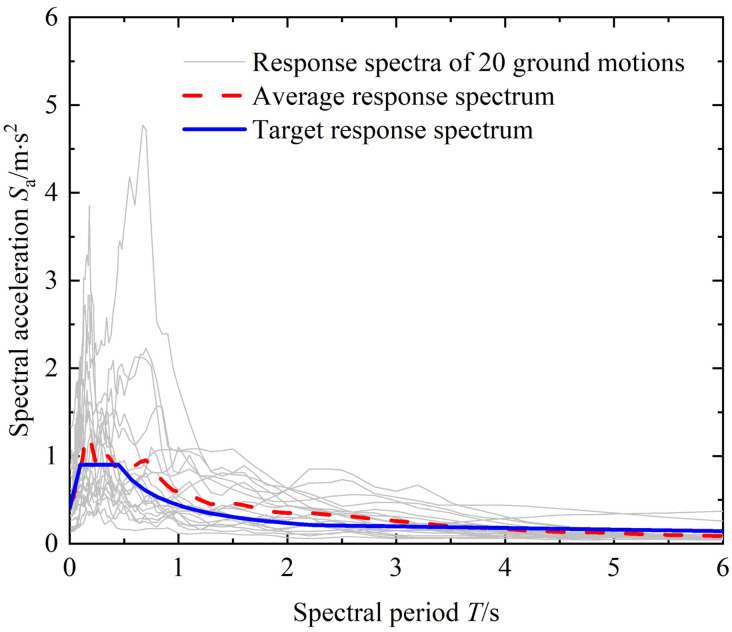
Ground motion response spectrum.

In selecting the ground motions, this study screened records from the PEER strong motion database. The selection procedure followed the structural design target spectrum specified in the Chinese standard [35], and the records were determined through response-spectrum-matching techniques. In addition, the findings of Shome and Cornell [36,37] indicate that, for mid-rise to high-rise buildings, using 10–20 ground-motion records are sufficient to achieve adequate accuracy in seismic performance assessment. Therefore, twenty ground-motion records were ultimately adopted in this study. Fig 10 presents a comparison between the average response spectrum of the selected ground motions and the design target spectrum. The average spectrum of the selected ground motions is in good agreement with the target design spectrum, which guarantees the reliability of the subsequent dynamic analysis. The reason for using spectral acceleration *S*_a_(*T*₁) (unit: m/s^2^) as the intensity measure is its proven efficiency and sufficiency in predicting structural seismic response at the fundamental period, which reduces dispersion in IDA results. Table 7 shows the basic information of ground motion.

**Table 7:** 20 basic information of ground motion.

I.D.	Name	Observation station	Event	Magnitude
1	TCU068-E	TCU068	Chi-Chi, Taiwan	7.62
2	LGP000	LGPC	Loma Prieta	6.93
3	NIG019EW	NIG019	Niigata, Japan	6.63
4	MYG005NS	MYG005	Iwate	6.90
5	JEN022	Jensen Filter Plant Administrative Building	Northridge-01	6.69
6	SCE011	Sylmar – Converter Sta East	Northridge-01	6.69
7	LFS270	Loleta Fire Station	Cape Mendocino	7.01
8	A-STU270	Sturno (STN)	Irpinia, Italy-01	6.90
9	STG090	Saratoga – Aloha Ave	Loma Prieta	6.93
10	ARE090	Arcelik	Kocaeli, Turkey	7.51
11	ABBAR—T	Abbar	Manjil, Iran	7.37
12	65010NS	Joetsu Kakizakiku Kakizaki	Chuetsu-oki	6.80
13	TCU068-N	TCU068	Chi-Chi, Taiwan	7.62
14	BAM-L	Bam	Bam, Iran	6.60
15	MYG004NS	MYG004	Iwate	6.90
16	BNH270	Bunker Hill FAA	Cape Mendocino	7.01
17	ABY090	Amboy	Hector Mine	7.13
18	ABY360	Amboy	Hector Mine	7.13
19	ABBAR—L	Abbar	Manjil, Iran	7.37
20	65057EW	Oguni Nagaoka	Chuetsu-oki	6.80

### 4.3. The limit state definition of structural performance

Appropriate performance limit states are defined to quantitatively assess the seismic behavior and damage progression of the prefabricated frame structure. In this study, performance levels are classified into five categories, namely intact, slight damage, moderate damage, severe damage, and collapse, which are commonly adopted in performance-based seismic assessment frameworks. Different performance limit states can be characterized using different evaluation indices, and their corresponding threshold values vary depending on the selected damage measure. According to existing experimental and theoretical investigations, critical values are adopted for both the developed damage index and the traditional inter-story drift ratio. The specific definitions of the performance limit states and their corresponding threshold ranges for different evaluation indicators are summarized in Table 8.

**Table 8:** Thresholds of different evaluation indicators [28].

Index	Basically intact	Slight damage	Moderate damage	Severe damage	Collapse
*D*_*k*_	0 ~ 0.1	0.1 ~ 0.25	0.25 ~ 0.4	0.4 ~ 0.8	>0.8
*θ*_max_	0 ~ 0.2%	0.2% ~ 1%	1% ~ 2%	2% ~ 10%	>10% or 0.2*K*_e_

Note: *K*_e_ is the slope of IDA curve in elastic stage.

### 4.4. Incremental dynamic analysis

**Fig 11:**
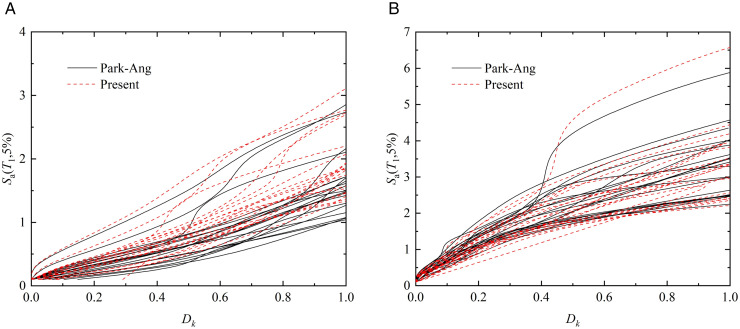
IDA curves of structures under different weighting methods based on *D*_*k*_ index. (a) IDA curve of prefabricated structure (b) IDA curve of cast-in-place structure.

**Fig 12:**
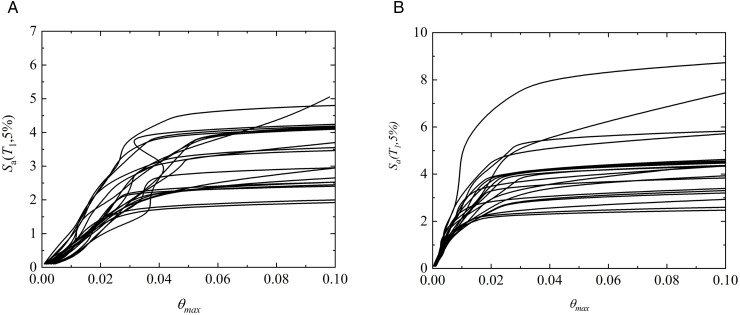
IDA curves of structures based on maximum inter-story drift ratio. (a) IDA curve of prefabricated structure (b) IDA curve of cast-in-place structure.

The termination criterion for IDA analysis strictly follows the collapse limit state defined in Table 8. When a two-parameter damage model is used as the engineering demand parameter, termination occurs when the damage index exceeds 0.8. When the maximum inter-story drift ratio is adopted as the engineering demand parameter, termination occurs either when its value exceeds 0.1 or when its slope drops below 20% of the elastic slope of the IDA curve. From the IDA curves shown in Figs 11 and 12, it can be observed that the structural responses exhibit relatively small dispersion in the elastic stage. As the seismic intensity increases and the structure enters the plastic stage, the discrepancies among different records gradually enlarge, which is attributed to the varying characteristics of the ground motions. Based on Figs 11 and 12, the IDA curves obtained using the two-parameter damage model with the damage index *D*_k_ exhibit significantly less dispersion than those based on the inter-story drift ratio. This indicates that a single displacement-based index is insufficient to accurately capture structural damage under different ground motions and may lead to notable errors. In addition, when the structures reach the collapse threshold, the seismic intensity corresponding to the demand parameters of the prefabricated frame is consistently lower than that of the cast-in-place frame, demonstrating that the seismic performance of the prefabricated system remains inferior.

As shown in Fig 11, when the proposed floor-weighted damage model is adopted, the IDA curves obtained using different weighting schemes exhibit similar global evolution trends, indicating that the damage assessment is not dominated by the choice of a specific weighting form. However, systematic differences can be observed between the Park–Ang weighting and the weighting strategy proposed in this study. Under the same seismic intensity level, before the onset of severe damage (i.e., *D*_k_ ≤ 0.4), the Park–Ang weighting generally yields larger global damage values than the proposed method. This is primarily because the Park–Ang formulation assigns relatively higher contributions to upper-story damage, which may overestimate the influence of damage accumulation in higher stories when the structural response is still governed by moderate nonlinear behavior.

Once the seismic demand rises sufficiently and the structure enters a severe damage state (*D*_k_ ≥0.4), an opposite trend is observed: the Park–Ang method tends to produce smaller damage values compared with the proposed weighting approach. This discrepancy arises because the Park–Ang weighting does not explicitly enhance the contribution of lower-story damage. Previous experimental observations and post-earthquake investigations [38,39] have consistently shown that, under strong seismic excitations, severe damage and collapse of frame structures are more likely to initiate at the lower stories, particularly at the structural base where deformation demand and cumulative energy dissipation tend to concentrate.

In contrast, the weighting method proposed in this study introduces a physically motivated modification that accounts for the variation of story importance along the height of the structure. Without altering the fundamental framework of the damage model, the proposed weighting emphasizes the critical role of lower stories during the progression of nonlinear damage, which is consistent with observed seismic damage patterns in frame structures. Therefore, the differences between the two weighting schemes reflect their respective sensitivities to damage distribution rather than a contradiction in damage definition, and the suggested approach offers a more reasonable depiction of damage progression under intense seismic inputs.

### 4.5. Characteristics of probabilistic seismic demand models

**Fig 13:**
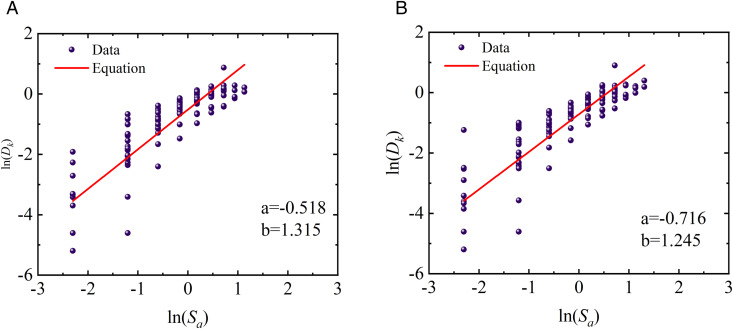
Probabilistic demand models of prefabricated structures based on different weighting schemes. (a) Two-parameter model weighted by Park-Ang (b) Two-parameter model weighted by the Present method.

**Fig 14:**
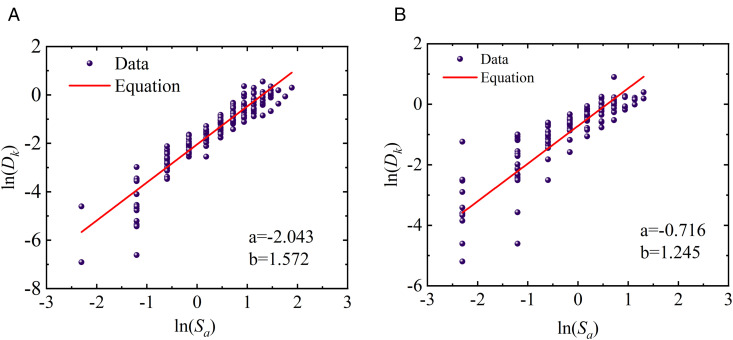
Probabilistic demand models of cast-in-place structures based on different weighting schemes. (a) Two-parameter model weighted by Park-Ang (b) Two-parameter model weighted by the Present method.

**Fig 15:**
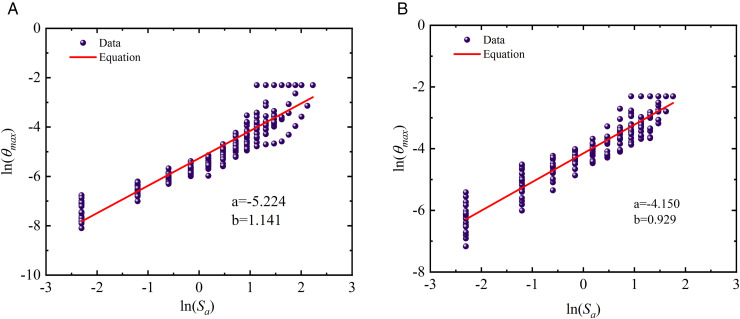
Probabilistic demand models of different structures based on maximum inter-story drift ratio. (c) cast-in-place structures (d) prefabricated structures.

Based on the IDA results presented in Fig 12 and Eq. (8), probabilistic seismic demand models (PSDMs) were further developed for fragility assessment. Specifically, the *EDP*s were defined using both the proposed two-parameter damage index *D*_*k*_ under different story-level weighting strategies and the conventional maximum inter-story drift ratio *θ*_max_. By fitting the relationships between the *IM* and the corresponding *EDP*s, PSDMs were established for each damage measure, providing a consistent probabilistic basis for comparing the effects of different damage-weighting schemes and drift-based demand definitions on the seismic fragility of the prefabricated and cast-in-place frames. The corresponding results are illustrated in Figs 13–15.

As observed from the probabilistic demand models presented in Figs 13 and 14, structural demands can be well fitted by the log-linear relationship ln(*D*)=*a* + *b*ln(*IM*), regardless of the two-parameter damage index *D*_*k*_ is adopted. This indicates that structural responses exhibit a stable power-law growth pattern with seismic intensity within the framework of IDA. Such a statistical law not only provides the necessary probabilistic basis for the subsequent calculation of fragility curves, but also demonstrates that the two-parameter damage index employed in this study possesses favorable modeling feasibility. In addition, Figs 13 and 14 reveal remarkable discrepancies in the fitted parameters *a* and *b* corresponding to different story-level damage weighting schemes, suggesting that the sensitivity of the global damage index *D*_*k*_ to seismic intensity is significantly affected by the weighting strategy. Specifically, the variation in the fitted slope *b* reflects the difference in the growth rate of global damage with increasing *IM*. A larger value of *b* implies a faster growth of the damage index under moderate-to-strong seismic intensities, indicating that the weighting scheme is more sensitive to the cumulative degradation effects during the plastic development stage. Since the global damage index is obtained by weighting story-level damage contributions, the aforementioned statistical differences indirectly reflect the varying emphasis of different weighting strategies on story damage contributions.

In light of the weighting formulation (Eq. (6)), the energy-dissipation-based weighting method proposed herein can automatically amplify the contribution of critical stories with concentrated plastic energy dissipation. Given that previous earthquake damage observations and analytical studies have commonly demonstrated that damage tends to concentrate in lower stories and govern the global instability mechanism under strong earthquakes, the proposed weighting method yields more reasonable statistical response characteristics during the rapid damage propagation stage. This provides a demand model foundation with stronger physical consistency for the subsequent collapse fragility assessment. Furthermore, for the two-parameter damage index *D*_*k*_ the demand growth slopes of cast-in-place frames (b = 1.572 and 1.479) are generally higher than those of prefabricated frames (b = 1.315 and 1.245). This indicates that cast-in-place structures undergo more pronounced stiffness degradation during elastoplastic development, and their damage demands are more sensitive to variations in seismic intensity. In contrast, prefabricated frame exhibits more gradual stiffness degradation due to relatively regular components and connection details, resulting in a lower growth rate of seismic demands. In the models using the maximum inter-story drift ratio *θ*_max_ as the demand parameter, the slope of cast-in-place structures is 1.141, while that of prefabricated structures is 0.929, which also confirms the faster growth of displacement demands in cast-in-place structures.

### 4.6. Seismic fragility analysis

**Fig 16:**
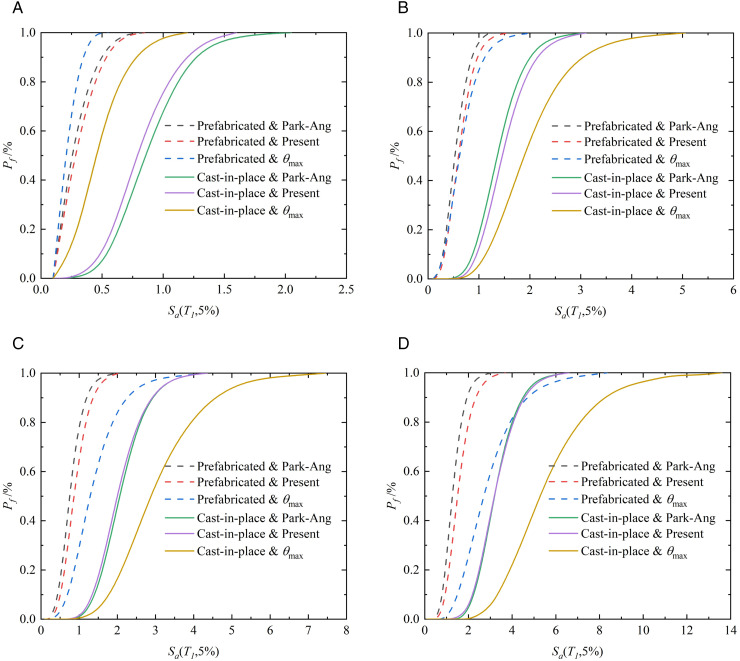
Seismic fragility curves of prefabricated structure and cast-in-place structure. (a) Slight damage (b) Moderate damage, (c) Severe damage (d) Collapse.

To further quantify the influence of different *EDP* on the evaluation results of structural collapse probability, Fig 16 compares the seismic fragility curves derived from the two-parameter damage model and the conventional inter-story drift ratio for both prefabricated and cast-in-place frame structures. Overall, the relative behavior of the two demand measures is clearly damage-state dependent. In the slight-damage state, the fragility curves based on the inter-story drift ratio exhibit higher exceedance probabilities than those obtained from the two-parameter damage index at the same ground-motion intensity. This phenomenon can be explained by the fact that structural response in this stage is dominated by elastic or near-elastic deformation, for which the peak inter-story drift ratio is highly sensitive. By contrast, the cumulative hysteretic energy dissipation remains limited, leading the two-parameter damage model to predict a comparatively lower damage exceedance probability.

As the seismic intensity increases and the structure enters the moderate-to-severe damage range, the trend gradually reverses. The fragility curves based on the two-parameter damage model shift toward lower intensity levels and rise more rapidly than those based on the inter-story drift ratio. Quantitative comparison at a spectral acceleration of 2.0 m/s^2^ further validates this trend: for the prefabricated frame, the collapse probabilities reach 95% using the Park-Ang weighted *D*_k_, 84% using the proposed weighted *D*_k_, but only 27% using *θ*_max_. It can be seen that using the maximum inter-story drift ratio underestimates the structural collapse probability by 68% and 57%, respectively. This is because the inter-story drift ratio cannot quantify damage behaviors such as structural energy dissipation, and it primarily reflects the collapse probability induced by nonlinear deformation at the top of the structure. For the cast-in-place frame, the corresponding probabilities are 4%, 5%, and nearly 0%, respectively. This highlights the increasing importance of cyclic degradation and cumulative energy dissipation in governing structural performance. In this stage, reliance on peak deformation alone becomes insufficient to characterize damage evolution, and the drift-based measure tends to underestimate the probability of exceeding higher damage states. In the severe-damage and collapse limit states, this discrepancy becomes more pronounced. The drift-only measure significantly overestimates the collapse resistance of both structural systems, whereas the two-parameter damage model provides a more conservative and physically realistic estimate of collapse risk. This is because the latter explicitly accounts for damage accumulation under repeated inelastic cycles, which is a key mechanism leading to instability under strong ground motions.

Further comparison between different damage-weighting strategies indicates that the proposed energy-dissipation-based story weighting method leads to higher exceedance probabilities in the severe-damage and collapse states than the Park–Ang weighting scheme. This difference is consistent with the IDA results discussed in Section 3.4. In the early damage stages, the Park–Ang weighting tends to yield larger damage values due to an overestimation of damage contributions from upper stories. However, as nonlinear response develops, damage progressively concentrates in the lower stories, where instability and collapse are more likely to initiate under strong earthquakes. Since the Park–Ang method does not explicitly enhance the contribution of lower-story damage, it may underestimate structural deterioration in advanced damage stages. In contrast, the proposed weighting method introduces a physically motivated modification that better reflects the vertical distribution of damage along the structural height, leading to more reasonable collapse risk estimates. significantly overestimates the collapse resistance. For all damage states and under all evaluation measures, the fragility curves of the prefabricated frame are consistently shifted toward lower intensity levels compared with those of the cast-in-place frame. The difference between the two systems is relatively small in the slight-damage state but becomes increasingly significant with damage progression, reaching its maximum in the collapse limit state.

## 5. Conclusions

This paper developed a damage-based seismic performance assessment method for full prefabricated frame structures and investigated the influence of damage demand measure and story-level weighting on IDA and fragility results. By integrating nonlinear dynamic analyses, damage characteristics, and probabilistic fragility assessment, the study reveals the limitations of conventional drift-based indices and clarifies the role of cumulative energy dissipation and damage distribution along the structural height in seismic fragility evaluation. The following main conclusions are drawn:

1) The proposed two-parameter damage model provides a more comprehensive and conservative seismic fragility assessment than the conventional inter-story drift ratio index.
2) Drift-based demand measures underestimate seismic fragility under strong earthquakes. Compared with the inter-story drift ratio, the two-parameter damage index consistently predicts higher damage exceedance probabilities at the same ground-motion intensity, especially in the moderate-to-collapse damage ranges. At a spectral acceleration of 2.0 m/s^2^, it can be seen that using the maximum inter-story drift ratio underestimates the structural collapse probability by 68% and 57%, respectively.
3) The prefabricated frame with steel plate hoop–bolt connections exhibit systematically higher seismic fragility than its cast-in-place counterpart under strong ground motions.
4) The story-level energy-dissipation weighting method offers a physically realistic improvement over existing weighting schemes for global damage aggregation.

In the final paragraph of the conclusions, the authors have added the limitations of the proposed method. The method imposes no restriction on the number of structural stories. However, since the simplified model used in the case study only addresses the prefabricated frame structure connected by bolts and steel plate hoops, its applicability and accuracy to other types of structures may be limited. These limitations have been supplemented in the revised manuscript.
